# Three-dimensional evaluation of soft tissues in hyperdivergent skeletal class II females in Guangdong

**DOI:** 10.1186/s12880-022-00782-w

**Published:** 2022-03-29

**Authors:** Xueqin Zhang, Jinxuan Zheng, Jianqing Deng, Zhaoxiang Wen, Zhengyuan Chen, Liyi Gan, Liping Wu

**Affiliations:** 1 Hospital of Stomatology, Sun Yat-sen University; Guangdong Provincial Key Laboratory of Stomatology; Guanghua School of Stomatology, Sun Yat-Sen University, Guangzhou, 510055 People’s Republic of China; 2 Department of Stomatology, Shenzhen Baoan Maternal and Child Health Hospital, Jinan University, Shenzhen, 518106 People’s Republic of China; 3 Department of Stomatology, The Eighth Affiliated Hospital Sun Yat-Sen University, Shenzhen, 518000 People’s Republic of China

**Keywords:** Three dimensional, Soft tissue analysis, Guangdong population, 3dMD face system

## Abstract

**Objectives:**

To establish the three-dimensional facial soft tissue morphology of adolescent and adult females in the Guangdong population and to study the morphological characteristics of hyperdivergent skeletal class II females in Guangdong compared with that of normodivergent class I groups.

**Materials and methods:**

The 3dMDface system was used to capture face scans of 160 patients, including 45 normal and 35 hyperdivergent skeletal class II adolescents (aged 11–14 years old) and 45 normal and 35 hyperdivergent skeletal class II adults (aged 18–30 years old). Thirty-two soft tissue landmarks were mapped, and 21 linear, 10 angular and 17 ratio measurements were obtained by 3dMDvultus analysis software. Data were assessed with a t-test of two independent samples between the normal adolescent and adult groups and between the normal and hyperdivergent skeletal class II groups.

**Results:**

The linear measurements of the Guangdong adult females were larger than those of the adolescents in both Class I and Class II groups. However, the angular and ratio measurements had no significant difference. The vertical linear measurements were higher and the sagittal and transverse linear measurements were smaller in the hyperdivergent class II group (*p* < 0.05). The soft tissue ANB angle, chin-lip angle, and mandibular angle were significantly larger and the soft tissue facial convexity angle and nasal convexity angle were significantly smaller in the hyperdivergent class II group (*p* < 0.05). Additionally, there were significant differences in the ratio measurements between the hyperdivergent class II groups and the control groups (*p* < 0.05).

**Conclusions:**

The three-dimensional facial morphology of Guangdong adolescent and adult females was acquired. The facial soft tissue measurements of the adults were higher in the three dimensions except for the facial convexity and proportional relationships which were similar, suggesting that the growth pattern remained the same. The three-dimensional facial soft tissue features of hyperdivergent skeletal class II were characterized by the terms “long, convex, and narrow”. Three-dimensional facial measurements can reflect intrinsic hard tissue characteristics.

**Supplementary Information:**

The online version contains supplementary material available at 10.1186/s12880-022-00782-w.

## Background

In recent years, more patients have sought orthodontic treatment to improve their facial aesthetics, and the majority have been female patients. Class II malocclusion has a high prevelance of 9.91% in Chinese schoolchildren [1]. Among the Guangdong population, skeletal class II malocclusion is quite common, and the chief complaint of these patients is facial protrusion [2]. Hyperdivergent skeletal class II malocclusion has obvious inharmonious jaw relationships in the sagittal, vertical and transverse directions. Fixing the facial soft tissue profile is the main treatment goal. The mechanism of vertical imbalance is complex. The growth pattern of the mandibular condyles with high-angle malocclusion is a backward growth, and the vertical growth type is expressed in the chin [3]. Clockwise rotation of the lower jaw due to the growth difference of anterior and posterior facial height is another important cause of the high-angle type [3]. These characteristics not only affect the patients’ appearance but also cause psychological and mental disorders [4].

Vertical problems affecting the sagittal direction and facial protrusion are the key challenges in the orthodontic treatment of hyperdivergent skeletal class II malocclusion [3]. Clinical treatment for hyperdivergent class II malocclusion is complex and involves extensive efforts to control vertical aspects while solving sagittal and horizontal problems and avoiding worsening the facial aesthetics. Orthodontic treatments changes hard tissues in three dimensions, followed by soft tissue changes. However, the changes in soft tissue and hard tissue might differ during treatment. The improvement of soft tissue can provide a visual impression of the treatment results to patients. Therefore, we should fully understand both soft and hard tissue facial features in three dimensions to accurately carry out the diagnostic analysis, treatment plan and result evaluation.

Traditional tools for facial soft tissue analysis are digital photos and frontal and lateral cephalometric radiographs [5]. However, limited information is obtained from these 2-D images. The accuracy and repeatability of these images are affected by the patients’ head position, camera angle, distance and so on. Three dimensional tools like cone beam computed tomography (CBCT) could overcome the drawbacks of 2-D images, but CBCT has radiation [6, 7]. Recently, three-dimensional surface imaging technology has been used to obtain three-dimensional facial soft tissue information, reveal facial soft tissue more intuitively, and make accurate measurements [8]. 3dMDface System (3dMD LLC, Atlanta, Ga.) is a 3D imaging device with high reproducibility and accuracy [9]. It can provide a basis for comprehensive diagnosis and treatment planning, with a wide range of clinical applications [10–12]. However, the three-dimensional facial soft tissue morphology of adolescent and adult females of the Guangdong population has not been well studied. Soft tissue changes are one of the patients’ objective requirements; therefore, research on soft tissue characteristics could aid in making comprehensive treatment plans. Few reports have examined the three-dimensional features of facial soft tissue in patients with hyperdivergent skeletal class II in Guangdong. In this study, we established a database of three-dimensional soft tissue characteristics of adolescent and adult hyperdivergent skeletal class II female patients compared to the characteristics of normal groups in Guangdong to treat this group of patients better, to lay the foundation for subsequent research, and to study the facial soft tissue characteristics of hyperdivergent skeletal class II female patients.

## Materials and methods

### Subjects

This study was approved by the Ethics Committee of Sun Yat-sen University. A total of 160 subjects were included in this study. All the subjects were selected from the patients who had treatment in dental hospital. 3-D photos were captured before their treatment. Guangdong population was confirmed via a self-administered questionnaire. The inclusion criteria of this study are shown in Table 1. The different groups were classified according to the reference range from Steiner’s analysis of Chinese population [13]. The exclusion criteria were facial asymmetry, previous orthodontic history, facial trauma or surgery.

**Table 1:** Grouping criteria

	Amount	Age (y old)	CVS stage	ANB angle (°)	GoGn-SN (°)
Group A	45	11–14	III or IV	0–5	27.3–37.7
Group B	45	18–30	Mature	0–5	27.3–37.7
Group C	35	11–14	III or IV	> 5	> 37.7
Group D	35	18–30	Mature	> 5	> 37.7

### 3dMD images

**Fig. 1:**
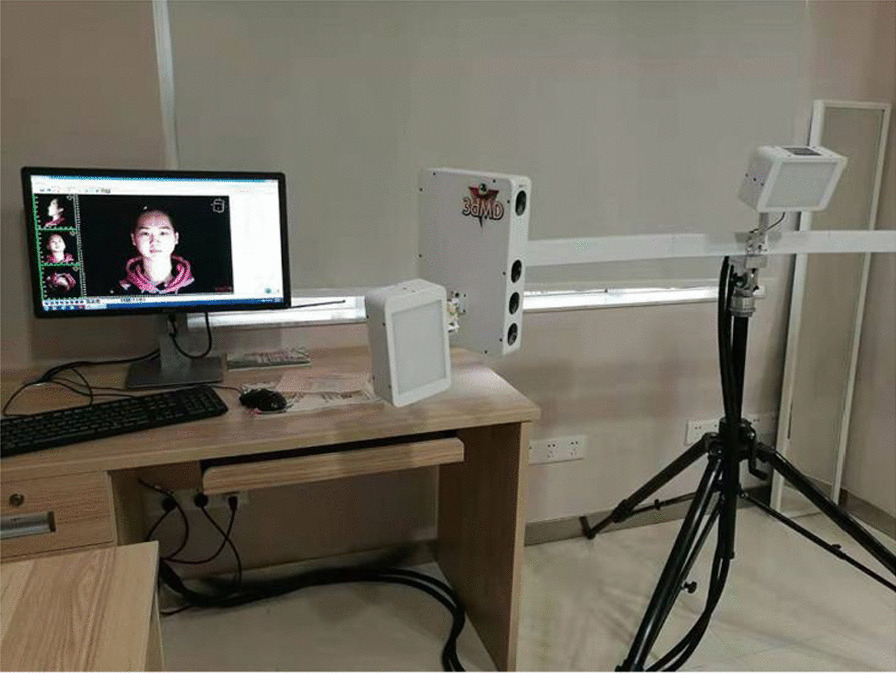
3dMD face system

The three-dimensional images were captured by a 3dMDface System (3dMD LLC, Atlanta, Ga.) and analyzed by 3dMDvultus software (3dMD LLC, Atlanta, Ga.)(Fig. 1). The 3dMD scanner had no radiation. The patient sat on an adjustable chair, and the distance between the subject and scanner was 1000 ~ 1100 mm. Before capturing the images, the instrument was calibrated according to the operating instructions. The system could automatically focus and capture a facial image of 180° between the ears. The capture speed was 1.5 ms [14]. The patient was seated in a relaxed, natural state, with both eyes looking straight ahead and a relaxed facial expression, keeping the posterior teeth at the maximum staggered position after swallowing, and the upper and lower lips closed gently. The data were stored in OBJ format files and imported into 3dMDvultus analysis software for further analysis.

### Parameters measured

**Fig. 2:**
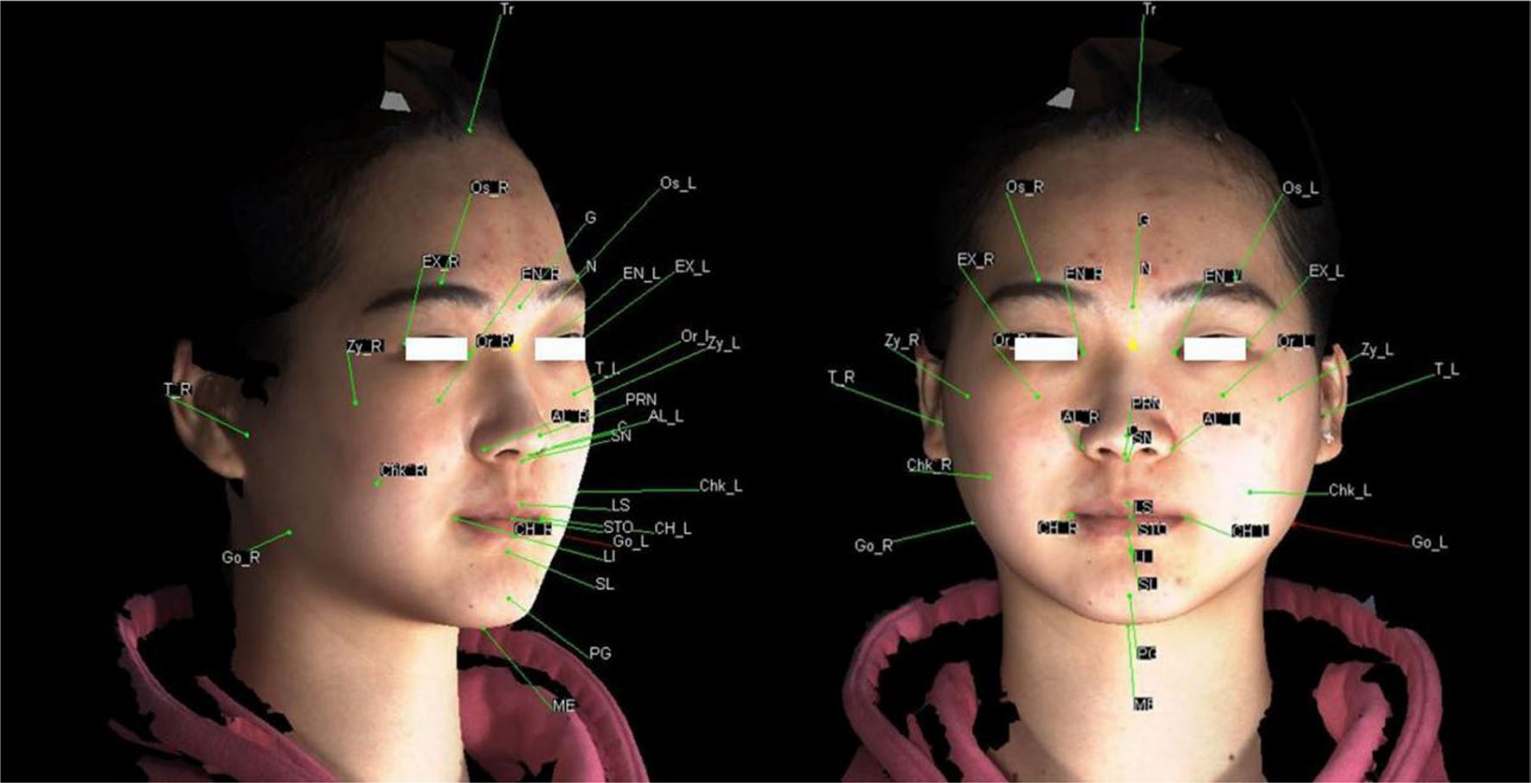
Soft tissue landmarks

3dMDvultus analysis software was applied to locate 32 soft tissue landmarks (Fig. 2) to calculate 21 linear, 10 angular and 17 ratio measurements (Tables 2, detailed measurement definition are seen in the Additional file 1). The three-dimensional facial soft tissue differences of normodivergent skeletal class I adolescent females were analyzed and compared with that of normodivergent skeletal class I adult females. 3D facial soft tissue discrepancies of the adolescents/adults with hyperdivergent skeletal class II malocclusion were analyzed and compared with that of normodivergent skeletal class I group.

**Table 2:** Landmarks

Trichion	Tr	Orbitale superius right	Os_R
Glabella	G	Orbitale superius left	Os_L
Nasion	N′	Orbitale right	Or_R
Pronasale	Prn	Orbitale left	Or_L
Columella	C	Cheek right	Chk_R
Subnasale	Sn	Cheek left	Chk_L
Labiale superius	Ls	Zygion right	Zy_R
Stomion	Sto	Zygion left	Zy_L
Labiale inferius	Li	Tragion right	Tra_R
Sublabiale	Sl	Tragion left	Tra_L
Pogonion	Pg′	Alare right	Al_R
Menton	Me′	Alare left	Al_L
Exocanthion right	Ex_R	Cheilion right	Ch_R
Exocanthion left	Ex_L	Cheilion left	Ch_L
Endocanthion right	En_R	Gonion right	Go_R
Endocanthion left	En_L	Gonion left	Go_L

### Statistical analysis

The operator was strictly trained to locate landmarks to ensure the landmarking process was accurate and consistent. All measurements in this study were performed by the same operator in a continuous period under the same conditions. If the difference was greater than 0.5 mm, the operator analyzed the reason and then reperformed the measurement until the distance between the two fixed points was not greater than 0.5 mm. This study used SPSS 20.0 software (IBM Corp., USA) for statistical analysis. Descriptive statistics of the measured data were acquired, and all statistical data are expressed as the mean ± standard deviation (x ± SD). A normal distribution of our data was shown using the normality test. A t-test of two independent samples was performed on the data of normodivergent skeletal class I adolescent and adult females and on the data of adolescents/adults with hyperdivergent skeletal class II malocclusion and the corresponding control group. α = 0.05 and *p* < 0.05 were considered statistically significant.

## Results

### Comparison of linear measurements between normodivergent skeletal class I adolescent and adult females

There were significant differences between these groups. In the vertical direction, the anterior facial height, anterior upper facial height and posterior facial height of the adult group were larger than those of the adolescent group (*p* < 0.05). Although the anterior forehead height, anterior lower facial height, lip height, mandibular height and chin height were larger than those of the adolescent group, the difference was not statistically significant. In the sagittal direction, the facial depth and mandible length in the adult group were larger than those in the adolescent group (*p* < 0.05). In the transverse direction, the facial width, mandibular width, interzygomatic width, buccal width, outer canthic diameter, lip width and nasal width of the adult group were larger than those of the adolescent group (*p* < 0.05). In summary, the facial three-dimensional distance measurements of the normodivergent skeletal class I adult female patients were larger than those of the adolescent group (Table 3).

**Table 3:** Linear measurements comparison between Guangdong normodivergent skeletal Class I adolescent and adult females

Linear measurements (mm)	Adolescent (n = 45)	Adult (n = 45)	t value	*P* value
	${\overline{\text{x}}}$ ± SD	${\overline{\text{x}}}$ ± SD		
Tr–G	61.69 ± 11.87	63.12 ± 7.67	0.679	0.499
N′–Me′	111.57 ± 5.02	113.80 ± 3.43	2.459	0.016*
N′–Sn	49.82 ± 2.91	50.88 ± 1.99	2.005	0.048*
Sn–Me′	64.36 ± 4.04	65.70 ± 2.74	1.836	0.070
Tra_R–Go′_R	47.56 ± 4.19	51.24 ± 3.35	4.603	0.000**
Tra_L–Go′_L	47.31 ± 5.10	51.10 ± 3.79	4.001	0.000**
Ls–Li	18.62 ± 2.11	18.83 ± 2.15	0.483	0.630
Sto–Me′	44.02 ± 3.73	44.38 ± 2.53	0.536	0.593
Sl–Me′	27.79 ± 3.67	28.60 ± 2.39	1.231	0.222
Sn–Tra_R	111.26 ± 4.59	113.03 ± 3.22	2.121	0.037*
Sn–Tra_L	110.76 ± 4.74	113.55 ± 3.12	3.290	0.001*
Go′–Me′_R	84.20 ± 5.01	88.07 ± 3.09	− 4.417	0.000**
Go′–Me′_L	84.19 ± 5.06	88.11 ± 3.18	− 4.402	0.000**
Tra_R–Tra_L	139.01 ± 5.53	142.33 ± 3.11	3.509	0.001*
Go′_R–Go′_L	115.30 ± 6.17	119.83 ± 4.34	4.019	0.000**
Zy_R–Zy_L	110.60 ± 5.30	114.15 ± 2.97	3.925	0.000**
Ck_R–Ck_L	94.98 ± 4.51	101.05 ± 2.59	7.828	0.000**
Ex_R–Ex_L	92.90 ± 5.02	94.81 ± 2.99	2.186	0.031*
En_R–En_L	35.01 ± 2.57	35.55 ± 1.84	1.149	0.254
Ch_R–Ch_L	44.59 ± 3.26	48.57 ± 2.73	6.288	0.000**
Al_R–Al_L	31.84 ± 2.37	32.94 ± 1.84	2.464	0.016*

*Represent *p* < 0.05; **represent *p* < 0.01

### Comparison of angular measurements between normodivergent skeletal class I adolescent and adult females

There were no significant differences in the angular measurements between these two groups (Table 4).

**Table 4:** Angular measurements comparison between Guangdong normodivergent skeletal Class I adolescent and adult females

Angular measurements (°)	Adolescent (n = 45)	Adult (n = 45)	t value	*P* value
	${\overline{\text{x}}}$ ± SD	${\overline{\text{x}}}$ ± SD		
G–N′–Pn	142.20 ± 5.00	142.96 ± 5.72	0.670	0.503
N′–Sn–Pg′	163.19 ± 4.14	163.84 ± 4.17	0.740	0.461
N′–Prn–Pg′	137.41 ± 3.68	136.51 ± 3.75	− 1.144	0.256
Sn–N–Sl	7.53 ± 2.30	7.68 ± 2.08	0.323	0.748
Prn–Sn–Ls	120.37 ± 9.87	120.68 ± 10.11	0.144	0.886
Li–Sl–Pg′	142.01 ± 12.79	141.73 ± 13.36	− 0.102	0.919
Go′_R–Pg′–Go′_L	78.76 ± 3.27	79.94 ± 4.61	1.405	0.164
Ls–Sto–Li	145.12 ± 10.95	143.07 ± 12.38	− 0.831	0.408
Tra_R–Go_R–Me′	133.21 ± 2.47	132.72 ± 3.37	0.789	0.432
Tra_L–Go_L–Me′	133.22 ± 2.57	132.91 ± 3.80	0.465	0.643

*Represent *p* < 0.05; **represent *p* < 0.01

### Comparison of ratio measurements between normodivergent skeletal class I adolescent and adult females

The lip width/lip height ratio and mandibular width/facial width ratio of the adult group were larger than those of the adolescent group, and the differences were statistically significant (*p* < 0.05). The outer canthic diameter/mandibular width of the adult group was smaller than that of the adolescent group, and the differences were statistically significant (*p* < 0.05). No significant differences in other ratio measurements were seen between these two groups (Table 5).

**Table 5:** Ratio measurements comparison between Guangdong normodivergent skeletal Class I adolescent and adult females

Ratio measurements (100%)	Adolescent (n = 45)	Adult (n = 45)	t value	*P* value
	${\overline{\text{x}}}$ ± SD	${\overline{\text{x}}}$ ± SD		
N′–Sn/N′–Me′	0.45 ± 0.02	0.45 ± 0.01	− 0.183	0.855
Sn–Me′/N′–Me′	0.58 ± 0.02	0.57 ± 0.02	− 0.688	0.493
N′–Sn/Sn–Me′	0.78 ± 0.08	0.77 ± 0.04	0.094	0.925
Sto–Me′/N′–Me′	0.39 ± 0.03	0.39 ± 0.02	− 1.594	0.114
Sl–Me′/N′–Me′	0.25 ± 0.03	0.25 ± 0.02	− 0.297	0.767
N′–Me′/Tra_R–Go′_R	2.30 ± 0.16	2.33 ± 0.15	0.983	0.351
N′–Me′/Tra_L–Go′_L	2.33 ± 0.15	2.33 ± 0.21	0.764	0.904
N′–Sn/Tra_R–Go′_R	0.99 ± 0.09	1.01 ± 0.07	− 1.309	0.447
N′–Sn/Tra_L–Go′_L	1.03 ± 0.12	1.01 ± 0.09	0.700	0.194
Sn–Me′/Tra_R–Go′_R	1.31 ± 0.09	1.30 ± 0.10	− 0.499	0.619
Sn–Me′/Tra_L–Go′_L	1.30 ± 0.11	1.31 ± 0.09	0.108	0.914
Ch_R–Ch_L/Ls–Li	2.42 ± 0.35	2.69 ± 0.29	3.930	0.000**
Ex_R–Ex_L/Tra_R–Tra_L	0.67 ± 0.03	0.66 ± 0.02	− 0.553	0.582
Ex_R–Ex_L/Go′_R–Go′_L	0.80 ± 0.04	0.73 ± 0.03	− 9.659	0.000**
Go′_R–Go′_L/Tra_R–Tra_L	0.83 ± 0.03	0.84 ± 0.02	2.071	0.041*
Sn–Tra_R/Sn–Tra_L	1.00 ± 0.02	1.00 ± 0.02	− 1.709	0.091
Go′–Me′_R/Go′–Me′_L	0.99 ± 0.04	0.99 ± 0.06	− 0.113	0.910

*Represent *p* < 0.05; **represent *p* < 0.01

### Comparison of linear measurements between adolescent females with hyperdivergent skeletal class II and those with normodivergent skeletal class I malocclusion

There were significant differences between the two groups. In the vertical direction, the anterior lower facial height and mandibular height of Class II group were larger, the posterior facial height of Class II group was smaller than that of control group, and the differences were statistically significant (*p* < 0.05). In the sagittal direction, the mandible length of the Class II group was smaller than that of the control group, and the differences were statistically significant (*p* < 0.05). In the transverse direction, the mandibular width, interzygomatic width and buccal width were smaller than those of the control group, and the differences were statistically significant (*p* < 0.05) (Table 6).

**Table 6:** Linear measurements comparison between hyperdivergent Class II and normodivergent Class I malocclusion of Guangdong adolescent females

Linear measurements (mm)	Hyperdivergent Class II (n = 35)	Normodivergent Class I (n = 45)	t value	*P* value
	${\overline{\text{x}}}$ ± SD	${\overline{\text{x}}}$± SD		
Tr-G	64.58 ± 9.38	61.69 ± 11.87	1.181	0.241
N′-Me′	112.67 ± 6.86	111.57 ± 5.02	0.829	0.409
N′-Sn	49.64 ± 3.98	49.82 ± 2.91	− 0.240	0.811
Sn-Me′	67.17 ± 4.69	64.36 ± 4.04	2.877	0.005*
Tra_R-Go′_R	45.81 ± 2.69	47.56 ± 4.19	− 2.145	0.035*
Tra_L-Go′_L	45.29 ± 2.66	47.31 ± 5.10	− 2.121	0.037*
Ls-Li	19.35 ± 2.44	18.62 ± 2.11	1.431	0.156
Sto-Me′	46.41 ± 4.13	44.02 ± 3.73	2.710	0.008*
Sl-Me′	29.16 ± 3.56	27.79 ± 3.67	1.679	0.097
Sn-Tra_R	110.05 ± 4.19	111.26 ± 4.59	− 1.219	0.226
Sn-Tra_L	109.60 ± 4.42	110.76 ± 4.74	− 1.120	0.266
Go′-Me′_R	80.58 ± 3.13	84.20 ± 5.01	3.741	0.000**
Go′-Me′_L	80.50 ± 3.26	84.19 ± 5.06	3.748	0.000**
Tra_R-Tra_L	136.81 ± 5.60	139.01 ± 5.53	− 1.757	0.083
Go′_R-Go′_L	111.06 ± 6.21	115.30 ± 6.17	− 3.042	0.003*
Zy_R-Zy_L	108.15 ± 5.25	110.60 ± 5.30	− 2.058	0.043*
Ck_R-Ck_L	92.17 ± 4.26	94.98 ± 4.51	− 2.837	0.006*
Ex_R-Ex_L	93.23 ± 3.55	92.90 ± 5.02	0.329	0.743
En_R-En_L	34.46 ± 2.32	35.01 ± 2.57	− 0.979	0.331
Ch_R-Ch_L	43.96 ± 3.79	44.59 ± 3.26	− 0.794	0.430
Al_R-Al_L	31.71 ± 2.53	31.84 ± 2.37	− 0.235	0.815

*Represent *p* < 0.05; **represent *p* < 0.01

### Comparison of angular measurements between adolescent females with hyperdivergent skeletal class II and those with normodivergent skeletal class I malocclusion

The soft tissue facial convexity angle and nasal convexity angle of the Class II group were smaller than those of the control group, and the differences were statistically significant (*p* < 0.05). The soft tissue ANB angle, chin-lip angle, and mandibular angle of the Class II group were larger than those of the control group, and the differences were statistically significant (*p* < 0.05) (Table 7).

**Table 7:** Angular measurements comparison between hyperdivergent Class II and normodivergent Class I malocclusion of Guangdong adolescent females

Angular measurements (°)	hyperdivergent Class II (n = 35)	normodivergent Class I (n = 45)	t value	*P* value
	${\overline{\text{x}}}$ ± SD	${\overline{\text{x}}}$ ± SD		
G-N′-Pn	144.33 ± 4.74	142.20 ± 5.00	1.936	0.057
N′-Sn-Pg′	156.19 ± 3.68	163.19 ± 4.14	− 7.874	0.000**
N′-Prn-Pg′	133.36 ± 3.55	137.41 ± 3.68	− 4.956	0.000**
Sn-N-Sl	9.77 ± 1.97	7.53 ± 2.30	− 4.590	0.000**
Prn-Sn-Ls	123.95 ± 8.29	120.37 ± 9.87	1.724	0.089
Li-Sl-Pg′	148.28 ± 11.41	142.01 ± 12.79	2.280	0.025*
Go′_R-Pg′-Go′_L	80.06 ± 3.68	78.76 ± 3.27	1.670	0.099
Ls-Sto-Li	147.30 ± 10.16	145.12 ± 10.95	0.909	0.366
Tra_R-Go_R-Me′	138.56 ± 2.96	133.21 ± 2.47	− 8.801	0.000**
Tra_L-Go_L-Me′	138.53 ± 2.99	130.22 ± 2.57	− 8.513	0.000**

*Represent *p* < 0.05; **represent *p* < 0.01

### Comparison of ratio measurements between adolescent females with hyperdivergent skeletal class II and those with normodivergent skeletal class I malocclusion

The anterior lower facial height/anterior facial height ratio, mandibular height/anterior facial height ratio, anterior facial height/posterior facial height ratio, anterior upper facial height/posterior facial height ratio, and anterior lower facial height/posterior facial height ratio of the Class II group were larger than those of the control group, and the differences were statistically significant (*p* < 0.05). The outer canthic diameter/facial width ratio and outer canthic diameter/mandibular width ratio of the Class II group were larger than those of the control group; the mandibular width/facial width of the Class II group was smaller than that of the control group (*p* < 0.05) (Table 8).

**Table 8:** Ratio measurements comparison between hyperdivergent Class II and normodivergent Class I of Guangdong adolescent females

Ratio measurements (100%)	Hyperdivergent Class II (n = 35)	Normodivergent Class I (n = 45)	t value	*P* value
	${\overline{\text{x}}}$ ± SD	${\overline{\text{x}}}$ ± SD		
N′-Sn/N′-Me′	0.44 ± 0.02	0.45 ± 0.02	− 0.758	0.451
Sn-Me′/N′-Me′	0.60 ± 0.02	0.58 ± 0.02	3.416	0.001*
N′-Sn/Sn-Me′	0.74 ± 0.07	0.78 ± 0.08	− 1.980	0.051
Sto-Me′/N′-Me′	0.41 ± 0.03	0.39 ± 0.03	3.124	0.003*
Sl-Me′/N′-Me′	0.26 ± 0.03	0.25 ± 0.03	1.614	0.111
N′-Me′/Tra_R-Go′_R	2.52 ± 0.17	2.30 ± 0.16	6.046	0.000**
N′-Me′/Tra_L-Go′_L	2.51 ± 0.16	2.33 ± 0.15	5.325	0.000**
N′-Sn/Tra_R-Go′_R	1.11 ± 0.09	0.99 ± 0.09	5.636	0.000**
N′-Sn/Tra_L-Go′_L	1.11 ± 0.09	1.03 ± 0.09	4.125	0.000**
Sn-Me′/Tra_R-Go′_R	1.46 ± 0.13	1.31 ± 0.09	6.100	0.000**
Sn-Me′/Tra_L-Go′_L	1.45 ± 0.15	1.30 ± 0.11	5.116	0.000**
Ch_R-Ch_L/Ls-Li	2.30 ± 0.31	2.42 ± 0.35	− 1.591	0.116
Ex_R-Ex_L/Tra_R-Tra_L	0.68 ± 0.03	0.67 ± 0.03	2.259	0.027*
Ex_R-Ex_L/Go′_R-Go′_L	0.84 ± 0.05	0.80 ± 0.04	3.079	0.003*
Go′_R-Go′_L/Tra_R-Tra_L	0.81 ± 0.03	0.83 ± 0.03	− 2.739	0.008*
Sn-Tra_R/Sn-Tra_L	1.00 ± 0.02	1.00 ± 0.02	− 0.157	0.875
Go′-Me′_R/Go′-Me′_L	0.99 ± 0.03	0.99 ± 0.04	− 0.032	0.974

*Represent *p* < 0.05; **represent *p* < 0.01

### Comparison of linear measurements between adult females with hyperdivergent skeletal class II and those with normodivergent skeletal class I malocclusion

In the vertical direction, the anterior facial height, anterior lower facial height, mandibular height, and chin height of the hyperdivergent skeletal class II group were greater than those of the control group (*p* < 0.05). In the sagittal direction, the left and right posterior facial height, left and right facial depth, and left and right mandibular body length were smaller than those of the control group. (*p* < 0.05). In the transverse direction, the facial width, mandibular width, interzygomatic width, buccal width, inner canthic diameter and nasal width were lower in the hyperdivergent skeletal class II group than in the control group (*p* < 0.05) (Table 9).

**Table 9:** Linear measurements comparison between hyperdivergent Class II and normodivergent Class I of Guangdong adult females

Linear measurements (mm)	Hyperdivergent Class II (n = 35)	Normodivergent Class I (n = 45)	t value	*P* value
	${\overline{\text{x}}}$ ± SD	${\overline{\text{x}}}$ ± SD		
Tr-G	61.49 ± 10.39	63.12 ± 7.67	− 0.807	0.422
N′-Me′	117.52 ± 5.01	113.80 ± 3.43	3.938	0.000**
N′-Sn	51.91 ± 2.68	50.88 ± 1.99	1.986	0.051
Sn-Me′	69.07 ± 4.15	65.70 ± 2.74	4.367	0.000**
Tra_R-Go′_R	47.48 ± 3.88	51.24 ± 3.35	4.642	0.000**
Tra_L-Go′_L	47.46 ± 3.80	51.10 ± 3.79	4.248	0.000**
Ls-Li	19.32 ± 2.30	18.83 ± 2.15	0.973	0.334
Sto-Me′	46.94 ± 3.74	44.38 ± 2.53	3.644	0.000**
Sl-Me′	30.44 ± 3.93	28.60 ± 2.39	2.593	0.011*
Sn-Tra_R	110.37 ± 4.87	113.03 ± 3.22	− 2.935	0.004*
Sn-Tra_L	111.06 ± 4.45	113.55 ± 3.12	− 2.934	0.004*
Go′-Me′_R	84.62 ± 3.86	88.07 ± 3.09	4.448	0.000**
Go′-Me′_L	84.59 ± 3.96	88.11 ± 3.18	4.410	0.000**
Tra_R-Tra_L	139.30 ± 4.59	142.33 ± 3.11	− 3.512	0.001*
Go′_R-Go′_L	115.55 ± 5.33	119.83 ± 4.34	− 3.952	0.000**
Zy_R-Zy_L	111.10 ± 3.96	114.15 ± 2.97	− 3.938	0.000**
Ck_R-Ck_L	94.90 ± 3.25	101.05 ± 2.59	− 9.428	0.000**
Ex_R-Ex_L	93.58 ± 3.07	94.81 ± 2.99	− 1.799	0.076
En_R-En_L	34.41 ± 1.95	35.55 ± 1.84	− 2.671	0.009*
Ch_R-Ch_L	47.63 ± 2.99	48.57 ± 2.73	− 1.469	0.146
Al_R-Al_L	31.15 ± 1.86	32.94 ± 1.84	− 4.296	0.000**

*Represent *p* < 0.05; **represent *p* < 0.01

### Comparison of angular measurements between adult females with hyperdivergent skeletal class II and those with normodivergent skeletal class I malocclusion

The soft tissue facial convexity angle and nasal convexity angle of the hyperdivergent skeletal class II adult female group in Guangdong were smaller than those of the control group. The soft tissue ANB angle, chin-lip angle, and left and right mandibular angle were larger than those of the control group, and the difference was statistically significant (*p* < 0.05) (Table 10).

**Table 10:** Angular measurements comparison between hyperdivergent Class II and normodivergent Class I of Guangdong adult females

Angular measurements (°)	Hyperdivergent Class II (n = 35)	Normodivergent Class I (n = 45)	t value	*P* value
	${\overline{\text{x}}}$± SD	${\overline{\text{x}}}$ ± SD		
G-N′-Pn	144.37 ± 4.82	142.96 ± 5.72	− 1.169	0.246
N′-Sn-Pg′	157.69 ± 3.67	163.84 ± 4.17	6.893	0.000**
N′-Prn-Pg′	132.97 ± 3.61	136.51 ± 3.75	4.257	0.000**
Sn-N-Sl	9.11 ± 1.84	7.68 ± 2.08	− 3.214	0.002*
Prn-Sn-Ls	121.54 ± 8.51	120.68 ± 10.11	− 0.406	0.686
Li-Sl-Pg′	148.83 ± 9.39	141.73 ± 13.36	− 2.674	0.009*
Go′_R-Pg′-Go′_L	79.53 ± 4.53	79.94 ± 4.61	0.397	0.692
Ls-Sto-Li	147.52 ± 9.65	143.07 ± 12.38	− 1.750	0.084
Tra_R-Go_R-Me′	137.76 ± 3.74	132.72 ± 3.37	− 6.313	0.000**
Tra_L-Go_L-Me′	137.73 ± 3.70	132.91 ± 3.80	− 5.694	0.000**

*Represent *p* < 0.05; **represent *p* < 0.01

### Comparison of ratio measurements between adult females with hyperdivergent skeletal class II and those with normodivergent skeletal class I malocclusion

There were significant differences in the facial soft tissue ratio measurements between hyperdivergent skeletal class II adult females and the corresponding normodivergent skeletal class I group in Guangdong. The anterior lower facial height/anterior facial height ratio, mandibular height/anterior facial height ratio, chin height/anterior facial height ratio, anterior facial height/posterior facial height ratio, anterior upper facial height//posterior facial height ratio, anterior facial height/posterior facial height ratio, and outer canthic diameter/mandibular width of the hyperdivergent skeletal class II group were significantly greater than those of the control group (*p* < 0.05). The anterior upper facial height/anterior lower facial height ratio, lip width/lip height ratio, and mandibular width/facial width ratio of the hyperdivergent skeletal class II group were smaller than those of the control group, and the difference was statistically significant (*p* < 0.05) (Table 11).

**Table 11:** Ratio measurements comparison between hyperdivergent Class II and normodivergent Class I of Guangdong adult females

Ratio measurements (100%)	Hyperdivergent Class II (n = 35)	Normodivergent Class I (n = 45)	t value	*P* value
	${\overline{\text{x}}}$ ± SD	${\overline{\text{x}}}$ ± SD		
N′-Sn/N′-Me′	0.45 ± 0.02	0.45 ± 0.01	0.078	0.938
Sn-Me′/N′-Me′	0.59 ± 0.02	0.57 ± 0.02	4.002	0.000**
N′-Sn/Sn-Me′	0.76 ± 0.51	0.77 ± 0.04	− 2.071	0.042*
Sto-Me′/N′-Me′	0.40 ± 0.02	0.39 ± 0.02	3.255	0.002*
Sl-Me′/N′-Me′	0.26 ± 0.03	0.25 ± 0.02	2.047	0.044*
N′-Me′/Tra_R-Go′_R	2.49 ± 0.20	2.33 ± 0.15	4.186	0.000**
N′-Me′/Tra_L-Go′_L	2.48 ± 0.19	2.33 ± 0.21	3.339	0.001**
N′-Sn/Tra_R-Go′_R	1.11 ± 0.09	1.01 ± 0.07	5.541	0.000**
N′-Sn/Tra_L-Go′_L	1.13 ± 0.07	1.00 ± 0.09	6.820	0.000**
Sn-Me′/Tra_R-Go′_R	1.46 ± 0.13	1.30 ± 0.10	6.583	0.000**
Sn-Me′/Tra_L-Go′_L	1.46 ± 0.13	1.31 ± 0.09	6.326	0.000**
Ch_R-Ch_L/Ls-Li	2.53 ± 0.35	2.69 ± 0.29	− 2.197	0.031*
Ex_R-Ex_L/Tra_R-Tra_L	0.67 ± 0.03	0.66 ± 0.02	1.633	0.129
Ex_R-Ex_L/Go′_R-Go′_L	0.81 ± 0.04	0.73 ± 0.03	9.733	0.000**
Go′_R-Go′_L/Tra_R-Tra_L	0.83 ± 0.03	0.84 ± 0.02	− 2.417	0.018*
Sn-Tra_R/Sn-Tra_L	0.99 ± 0.03	1.00 ± 0.02	− 0.418	0.631
Go′-Me′_R/Go′-Me′_L	0.99 ± 0.04	0.99 ± 0.06	− 0.307	0.760

*Represent *p* < 0.05; **represent *p* < 0.01

## Discussion

Patients of different races, regions, genders, ages, and malocclusion types have different soft and hard tissues [15–18]. If different population standards are directly applied to the evaluation of the Chinese population, the results could be biased. Bishara et al. studied the longitudinal changes in the facial soft tissue protrusions of 35 subjects from 5 to 45 years old and found that facial protrusion of men and women showed a decreasing trend with age [19]. Therefore, we need to establish reference standards for the corresponding races, regions, genders, ages, and malocclusion types to better serve the local population.

The peak of female facial changes occurs in years 10–15[19]. The 11- to 14-year-old females selected in this study were in a rapid growth change period. There were significant differences compared to adulthood, indicating that there was rapid growth in the three-dimensional facial linear measurements consistent with physiological age. The adult females’ anterior facial height, anterior upper facial height, facial depth, mandible length, facial width, mandibular width, intercondylar width, buccal width, outer canthic diameter, lip width and nasal width were larger than those of adolescents, indicating that the soft tissue development of 11- to 14-year-old adolescents has great growth potential with a higher, deeper and wider tendency. Angular measurements can reflect the relative protrusion of each part of the face. Our data revealed that the normodivergent skeletal class I adolescent and adult females had similar relative protrusions of the various parts of the face despite their age difference. Growth patterns are proportional relationships that change over time, and some longitudinal studies have shown that the craniofacial growth of both Class I and Class II subjects is similar [20, 21]. Most of the proportional measurements were similar in adolescent and adult groups, suggesting that from adolescence to adulthood, their growth patterns remained unchanged. The ratio of lip width/lip height in the adult group is larger than that in the adolescent group, which may be due to the lip thinning with age; the ratio of outer canthic diameter/mandibular width in the adult group is significantly smaller than that of adolescent group, and the ratio of mandibular width/facial width in the adult group is larger than that of adolescent group. The width of the mandible is still increasing due to growth and development.

Palomo et al. [22] found that compared with Class I girls, Class II girls had a longer facial pattern and more protrusive maxilla. The three-dimensional facial soft tissue measurements of adolescent females in Guangdong can reflect their distinctive characteristics of hyperdivergent skeletal class II, showing "long, convex, narrow" characteristics. The facial protrusion angle and nasal protrusion angle of hyperdivergent class II were smaller than those of the control group, and the soft tissue ANB angle and the chin-lip angle were significantly larger than those of the control group. The mandible is in the distal position relative to the forehead, nose, maxilla and upper lip, which reflects the problem of poor lower jaw development. The ratio measurement results may be due to the clockwise rotation of the mandible of the hyperdivergent patients or insufficient development of the mandibular ramus, which results in an increase in the height of the facial lower part and an increase in the ratio of the anterior height/posterior height. The soft tissue also showed a vertical growth pattern consistent with that of hard tissue.

Lateral cephalometrics has limitations in analyzing transverse problems, but the 3dMD system has the advantages of transverse width analysis. In this study, the mandibular width, interzygomatic width and buccal width were significantly smaller in hyperdivergent class II patients, which means they had deficiency in transverse development, and the facial width and maxillary width deficiency affected vertical and sagittal development of the lower part of the face.

Our results remind us of the strategies for the treatment of class II hyperdivergent patients. Early treatments of this type of adolescent patient may contribute to less complicated treatment or no surgery in adulthood. According to the facial characteristics of hyperdivergent skeletal class II adolescent patients, specific attention should be given during the expansion of the maxilla and upper arch and the coordinated width of the upper and lower arches. If the upper arch is narrow, maxillary expansion might be appropriately performed to create space for the mandible to move forward without increasing the anterior lower facial height and facial convexity. For hyperdivergent skeletal class II adolescent patients, growth potential might be of use to achieve orthopedic effects without surgery or to avoid more complicated treatments in adulthood. For hyperdivergent skeletal class II adolescents, high-pull headgear combined with functional appliances such as Herbst appliances, Bionator or twin-block appliances can be used to suppress the height of the upper and lower alveolar bones, relatively promote the forward growth of the mandible, and then counterclockwise rotate the mandible [23]. Orthodontic camouflage treatment could be applied to patients with mild to moderate skeletal discrepancies. For adult patients with severe skeletal discrepancies, orthognathic surgery might be needed [24, 25].

In conclusion, from adolescence to adulthood, facial soft tissue grew in three dimensions but maintained the same growth pattern in class II hyperdivergent patients. The three-dimensional soft tissue of hyperdivergent skeletal class II females was characterized as "long, convex, narrow", which was similar to a previous study of the hard tissue characteristics of hyperdivergent skeletal class II patients. Three-dimensional facial soft tissue measurement could reflect its intrinsic hard tissue features.

## Supplementary Information

**Additional file 1**. Detailed measurement definition.

## Data Availability

All data generated or analysed during this study are included in this published article and its supplementary files.
