# Supplementary material for: Three-dimensional evaluation of soft tissues in hyperdivergent skeletal class II females in Guangdong
Source: BMC Med Imaging. 2022 Mar 29;22:56. doi: 10.1186/s12880-022-00782-w (PMC8966193; doi:10.1186/s12880-022-00782-w)
Supplement: Supplementary file 1 — Additional file 1. Detailed measurement definition. [file 12880_2022_782_MOESM1_ESM.docx]

Additional file 1

| Additional file 1: Table S1. Linear measurement definition | |
| --- | --- |
| Tr-G  N’-Me’  N’-Sn  Sn-Me’  Tra_R-Go’_R/Tra_L-Go’_L  Ls-Li  Sto-Me’  Sl-Me’  Sn-Tra_R/Sn-Tra_L  Go’-Me’_R/Go’-Me’_L  Tra_R-Tra_L  Go’_R-Go’_L  Zy_R-Zy_L  Ck_R-Ck_L  Ex_R-Ex_L  En_R-En_L  Ch_R-Ch_L  Al_R-Al_L | anterior forehead height  anterior facial height  anterior upper facial height  anterior lower facial height  Right/left posterior facial height  Lip height  mandibular height  chin height  Right/left facial depth  Right/left mandible length  facial width  mandibular width  inter-zygomatic width  buccal width  outer canthic diameter  Inner canthic diameter  lip width  nasal width |

| Additional file 1: Table S2. Angular measurement definition | |
| --- | --- |
| G-N’-Prn  N’-Sn-Pg’  N’-Prn-Pg’  Sn-N’-Sl  Prn-Sn-Ls  Li-Sl-Pg’  Go’_R-Pg’-Go’_L  Ls-Sto-Li  Tra_R-Go_R-Me’/Tra_L-Go_L-Me’ | nasal frontal angle  soft tissue facial convexity angle  nasal convexity angle  soft tissue ANB angle  nasal convexity angle  chin-lip angle  mandibular convexity angle  lip angle  Right/left mandibular angle |

| Additional file 1: Table S3. Ratio measurement definition | |
| --- | --- |
| N’-Sn/N’-Me’  Sn-Me’/N’-Me’  N’-Sn/Sn-Me’  Sto-Me’/N’-Me’  Sl-Me’/N’-Me’  N’-Me’/Tra_R-Go’_R  N’-Me’/Tra_L-Go’_L  N’-Sn/Tra_R-Go’_R  N’-Sn/Tra_L-Go’_L  Sn-Me’/Tra_R-Go’_R  Sn-Me’/Tra_L-Go’_L  Ch_R-Ch_L/Ls-Li  Ex_R-Ex_L/Tra_R-Tra_L  Ex_R-Ex_L/Go’_R-Go’_L  Go’_R-Go’_L/Tra_R-Tra_L  Sn-Tra_R/Sn-Tra_L  Go’-Me’_R/Go’-Me’_L | anterior upper facial height/anterior facial height  anterior lower facial height/anterior facial height  anterior upper facial height/anterior lower facial height  mandibular height/anterior facial height  chin height/anterior facial height  anterior facial height/right posterior facial height  anterior facial height/left posterior facial height  anterior upper facial height/right posterior facial height  anterior upper facial height/left posterior facial height  anterior lower facial height/right posterior facial height  anterior lower facial height/left posterior facial height  lip width/lip height  outer canthic diameter/facial width  outer canthic diameter/mandibular width  mandibular width/facial width  Right facial depth/left facial depth  Right mandible length/left mandible length |

| Additional file 1: Table S4. Intraexaminer Correlation Coefficients | | | |
| --- | --- | --- | --- |
| Landmarks | X | Y | Z |
| Tr | 0.996 | 1.000 | 1.000 |
| G | 1.000 | 0.997 | 1.000 |
| N’ | 1.000 | 1.000 | 1.000 |
| Prn | 1.000 | 1.000 | 0.999 |
| C | 1.000 | 1.000 | 1.000 |
| Sn | 1.000 | 1.000 | 1.000 |
| Ls | 1.000 | 1.000 | 1.000 |
| Sto | 1.000 | 1.000 | 1.000 |
| Li | 1.000 | 1.000 | 1.000 |
| Sl | 1.000 | 0.998 | 1.000 |
| Pg’ | 1.000 | 1.000 | 1.000 |
| Me’ | 0.998 | 1.000 | 1.000 |
| Ex_R | 1.000 | 1.000 | 1.000 |
| Ex_L | 1.000 | 1.000 | 1.000 |
| En_R | 1.000 | 1.000 | 1.000 |
| En_L | 1.000 | 1.000 | 1.000 |
| Os_R | 1.000 | 1.000 | 1.000 |
| Os_L | 1.000 | 1.000 | 1.000 |
| Or_R | 1.000 | 1.000 | 1.000 |
| Or_L | 1.000 | 1.000 | 1.000 |
| Chk_R | 0.998 | 1.000 | 0.999 |
| Chk_L | 0.989 | 0.997 | 1.000 |
| Zy_R | 1.000 | 1.000 | 0.999 |
| Zy_L | 1.000 | 0.998 | 0.999 |
| Tra_R | 1.000 | 1.000 | 1.000 |
| Tra_L | 1.000 | 1.000 | 1.000 |
| Al_R | 1.000 | 1.000 | 1.000 |
| Al_L | 1.000 | 1.000 | 1.000 |
| Ch_R | 1.000 | 1.000 | 1.000 |
| Ch_L | 1.000 | 1.000 | 1.000 |
| Go_R | 0.983 | 0.998 | 1.000 |
| Go_L | 0.999 | 0.999 | 0.999 |
